# The C-reactive protein to lymphocyte ratio and 28-day mortality in critically ill patients with sepsis: a retrospective propensity score–matched cohort study

**DOI:** 10.1515/med-2026-1548

**Published:** 2026-11-11

**Authors:** YuanWen Jing, ZhiWei Su, JiaQi Tang, HongGuang Ding, Hongke Zeng

**Affiliations:** Department of Critical Care Medicine, Guangdong Provincial People’s Hospital, Guangdong Academy of Medical Sciences, Southern Medical University, Guangzhou,Guangdong, China; Department of Emergency Medicine, The Third Affiliated Hospital of Southern Medical University, Guangzhou, Guangdong, China; Department of Intensive Care Medicine, Fifth Affiliated Hospital of Guangzhou Medical University, Guangzhou, 510080, Guangdong, China; Department of Emergency Medicine, Guangdong Provincial People’s Hospital, Guangdong Academy of Medical Sciences, Southern Medical University, Guangzhou, Guangdong, China

**Keywords:** C-reactive protein to lymphocyte ratio, sepsis, mortality, MIMIC-IV database, biomarker

## Abstract

**Objectives:**

This investigation aimed to delineate the association between the C-reactive protein to lymphocyte ratio (CLR) and all-cause mortality in adults sepsis patients.

**Methods:**

Drawing on information from the Medical Information Mart for Intensive Care (MIMIC)-IV database, release 3.0, this research assessed the CLR by factoring in C-reactive protein (CRP) and lymphocyte. Patients with sepsis are identified based on the diagnostic criteria of sepsis-3. The comorbidities are identified using the coding of the International Classification of Diseases (ICD). The investigation identified the pivotal CLR level impacting patient survival using x-tile. To address potential confounding factors, a 1:1 propensity score matching (PSM) method was utilized. The main focus was the 28-day mortality of sepsis patients. Kaplan–Meier curves were used to compare survival probabilities across groups. It then examined the effects on all cause mortality through multivariate Cox proportional hazards regression models. Subsequently, restricted cubic spline (RCS) methods were applied to delve into the linear or nonlinear nature CLR of the relationship between CLR and all cause mortality, with further insights gained from interaction and subgroup analyses. Furthermore, The predictive capacity of was assessed via ROC curve analysis and subgroup analysis.

**Results:**

The cohort comprised 1,321 sepsis patients. Post-PSM,analysis involved 300 matched patient pairs. Adjusted Cox proportional hazard models indicated a significant association of high CLR (≥228.5) with increased 28-day all cause mortality (HR=1.51, 95 % CI: 1.18–1.93, p<0.001). Associations of high CLR with elevated risk were consistent across 28-day, 60-day, 90-day, ICU and in hospital all cause mortality. Subsequent RCS analysis post-PSM underscored a negative nonlinear relationship between CLR and all cause mortality over terms, without significant interaction effects across all different subgroups for all cause mortality. In addition, combining CLR with disease severity scores enhances the predictive value for 28-day mortality.

**Conclusions:**

In critically ill patients suffering from sepsis, an elevated CLR upon admission correlated with a rise in all-cause mortality at various stages, including the 28-day, 60-day, 90-day, ICU and in-hospital mortality, solidifying its position as an independent mortality risk determinant.

## Introduction

Sepsis, a dysregulated host response to infection culminating in life-threatening organ dysfunction, continues to pose substantial challenges in critical care medicine [1], 2]. With an estimated global incidence exceeding 48 million cases annually and mortality rates ranging from 30 to 50 % depending on disease severity and healthcare settings, this complex syndrome remains the leading cause of death intensive care units [3]. The pathophysiological heterogeneity of sepsis and the lack of reliable prognostic biomarkers have significantly hindered the development of personalized therapeutic strategies, underscoring the urgent need for robust predictive tools that integrate both inflammatory and immunological parameters.

C-reactive protein (CRP), a pentameric acute-phase reactant synthesized by hepatocytes under interleukin-6 stimulation, has emerged as a cornerstone biomarker in sepsis diagnostics. Its serum concentration, which can increase up to 1,000-fold within 24–48 h of inflammatory challenge, reflects the intensity of systemic inflammation [4]. Conversely, lymphocytopenia, a hallmark of sepsis-induced immunosuppression, demonstrates strong correlation with disease severity and adverse outcomes [5]. The C-reactive protein to lymphocyte ratio (CLR), by synergistically combining these complementary biomarkers, may provide enhanced prognostic value through simultaneous evaluation of both pro-inflammatory drive and adaptive immune competence. This dual parameter approach addresses critical limitations of unidimensional biomarkers in capturing the complex immunopathology of sepsis.

Emerging evidence suggests CLR’s potential clinical utility in various inflammatory conditions. In COVID-19 pneumonia, CLR demonstrated superior predictive accuracy for disease severity compared to standalone CRP or lymphocyte counts [6], 7]. Similar prognostic associations have been observed in acute pancreatitis and post-cardiac surgery patients [8], 9]. However, the diagnostic and prognostic significance of CLR in sepsis remains underexplored.

This study leverages the Medical Information Mart for Intensive Care IV (MIMIC-IV) database (v3.0), a comprehensive repository containing high-resolution clinical data from 94,458 ICU admissions at a tertiary academic medical center, to address these knowledge gaps [10]. This study aims to investigate the correlation between CLR and prognosis in patients with sepsis using data from the MIMIC-IV database (version 3.0). By analyzing the CLR and its association with clinical outcomes, we hope to provide further evidence on its potential as a prognostic marker in sepsis and contribute to the development of more effective predictive models for sepsis management.

## Materials and methods

### Source of data

This investigation was conducted through a retrospective cohort approach, leveraging the Medical Information Mart for Intensive Care (MIMIC-IV) database (version 3.0). Recognized as a critical asset in critical care research, MIMIC-IV provides an extensive collection of anonymized clinical data related to ICU patient care. It features an enhanced and updated compilation of clinical variables ranging from demographic details to comprehensive physiological data and therapeutic measures. Esteemed as one of the most extensive and widely utilized databases in the realm of intensive care medicine, MIMIC-IV furnishes essential assets for analytical and research endeavors. It is instrumental for the exploration of outcomes in critical care, the development of predictive models, support for clinical decision-making, and a variety of other scholarly activities. Permission to access and use the MIMIC-IV database for this study was obtained from both the Massachusetts Institute of Technology and the Institutional Review Board at Beth Israel Deaconess Medical Center.

### Ethical considerations and data privacy

In adherence to ethical guidelines and to preserve patient confidentiality, this study utilized data that was rigorously de-identified, ensuring all patient information remained confidential. ZhiWei Su, obtained certification to access the MIMIC-IV database by completing the “Protecting Human Research Participants” online course provided by the National Institutes of Health (Record ID: 68674529), demonstrating compliancewith necessary ethical standards for research involving human subjects. Prior to data extraction, ZhiWei Su received specialized training to ensure conformity with established research protocols and methodologies. The research team meticulously crafted a series of data extraction procedures, which were preliminarily tested to refine their precision and feasibility. Furthermore, to verify the data’s reliability, the study incorporated multiple validation strategies, including an independent audit of essential data points and the utilization of statistical software for conducting consistency evaluations, thereby identifying and rectifying any discrepancies or errors. This study used the publicly available, de-identified MIMIC-IV database, which was approved by the relevant IRBs, and informed consent was waived.

### Population of the study and extraction of variables

The focal point of this analysis was the initial blood routine conducted upon ICU admission, designated as the primary exposure variable. The treatment measures mainly involve whether any such treatment was administered during the entire period of ICU hospitalization. Data extraction was performed from the MIMIC-IV database, utilizing SQL queries within a PostgreSQL environment. The extraction encompassed five key areas (with the missing proportions shown on the right): demographic information [including Age, Gender, Race, and weight (0.1 %)], comorbid conditions [Myocardial infarction, Congestive heart failure, Peripheral vascular disease, Cerebrovascular disease, Dementia, Chronic lung disease, Rheumatism, Peptic ulcer disease, Liver disease, Diabetes, Paraplegia, Kidney disease, Malignant tumor, hypertension, Chronic obstructive pulmonary disease (COPD)], Vital statistics [heart rate, Systolic Blood Pressure (SBP), Diastolic Blood Pressure (DBP), Temperature (0.1 %), and Respiratory rate (RR)], Laboratory findings [(white blood cell counts (WBC), Platelets, Neutrophils, Lymphocytes, Hematocrit, Red cell volume distribution width (RDW) (0.1 %), Bilirubin total (6.7 %), Creatinine, Blood urea nitrogen (BUN), Albumin (ALB) (18 %), Aspartate Aminotransferase (AST) (5.5 %), Bicarbonate, Partial pressure of oxygen (PO2) (10.4 %), Potential of hydrogen (PH) (10.5 %), Partial thromboplastin time (PTT) (1.2 %), International normalized ratio (INR) (1.1 %)],Clinical severity score [Glasgow coma score (GCS), Sequential organ failure assessment (SOFA) score, Simplified acute physiology score-II (SAPS-II), Acute physiology score-III (APS-III)],Treatments [Ventilator, Renal replacement treatment (RRT), Vasopressor]. The proportion in parentheses represents the missing proportion of that variable.

To ensure the integrity of the data, variables with a missing value percentage exceeding 20 % are excluded. For variables with a missing data proportion lower than 20 %, the multiple imputation method will be adopted to fill in the missing values multiple times using other available data points [11]. Multiple imputation was performed using SPSS Statistics 26. We generated m=5 imputed datasets using the Predictive Mean Matching method. All variables used in the main analysis (including outcome indicators and CLR) were included in the imputation model. Pooled estimates were obtained according to Rubin’s rules. The specific diagrams can be found in Supplementary Images 1 and 2. The variables that were excluded due to more than 20 % missing values or excessive collinearity are listed in Supplementary Table 1. This study also used the VIF method to confirm that the collinearity among all variables is less than 5, thereby enhancing the reliability of the data results [12]. This can be clearly seen in Figure 1.

**Figure 1:**
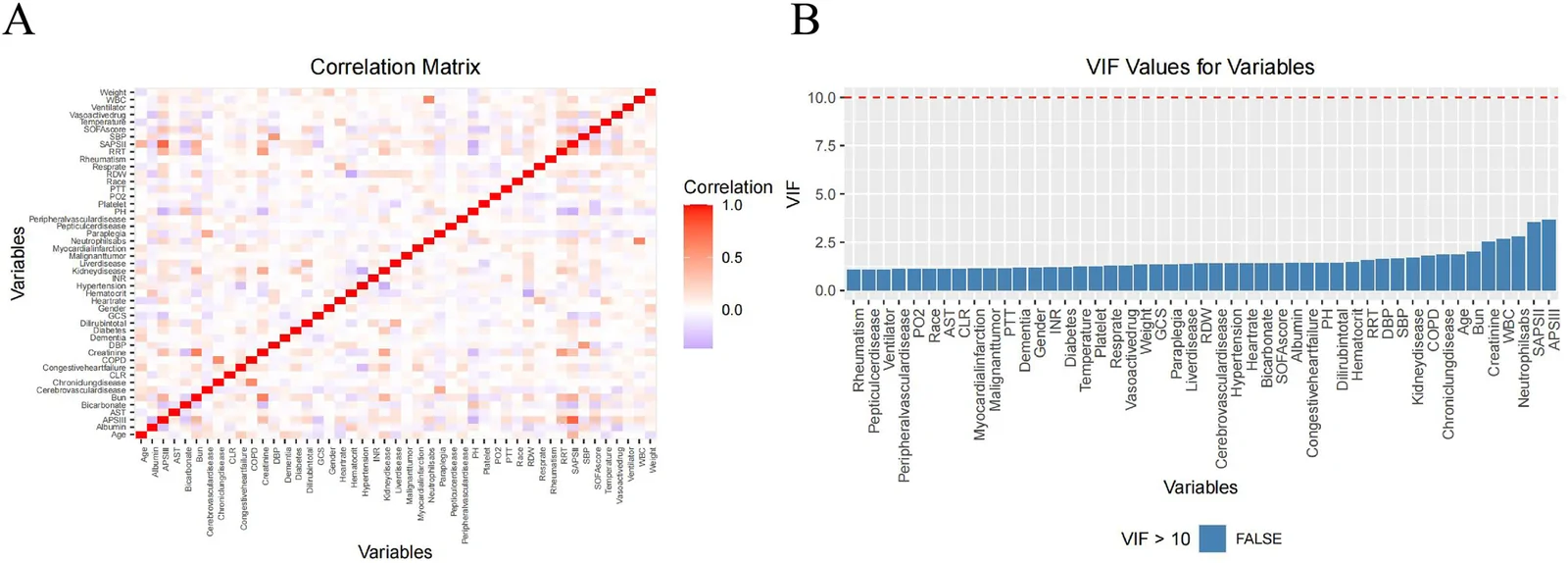
The VIF values of the variables and the analysis of the correlation matrix. (A) Correlation matrix between variables. (B) The VIF values of each variable.

Figure 2 presents the flowchart for the selection procedure of the study population. The diagnosis of sepsis was based on the Third International Consensus Definitions for Sepsis and Septic Shock (Sepsis-3). Of these, during this process, 27,035 patients were excluded because they were repeatedly admitted to the ICU or stayed in the ICU for less than 24 h. As a result, the number of research subjects was reduced to 67,423. Furthermore, according to the research design, non-sepsis patients (39,862 cases),patients without lymphocyte detection (11,840 cases),and patients without CRP detection (14,400 cases) were further excluded. Following these criteria, a cohort of 1,321 eligible patients was identified for further analysis. Then, using the x-tile software, the patients were stratified based on the CLR score (Figure 3). Among them, 988 patients had a CLR score lower than 228.5,and 333 patients had a CLR score of 228.5 or higher. The propensity score matching method was employed to balance the two groups. In the final analysis, there were 300 patients in each layer, as shown in Figure 2.

**Figure 2:**
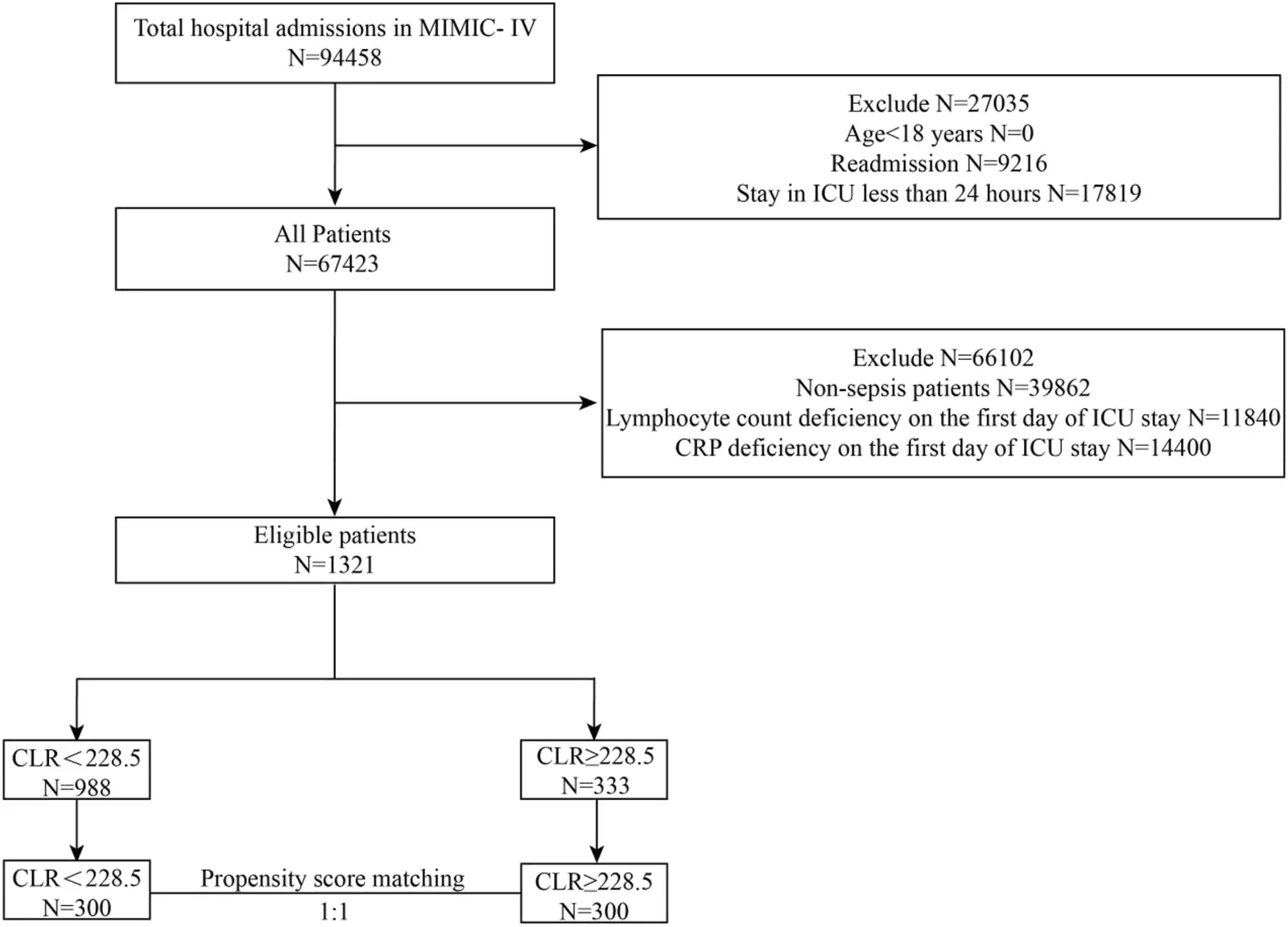
Flowchart of select study population from the MIMIC-IV database.

**Figure 3:**
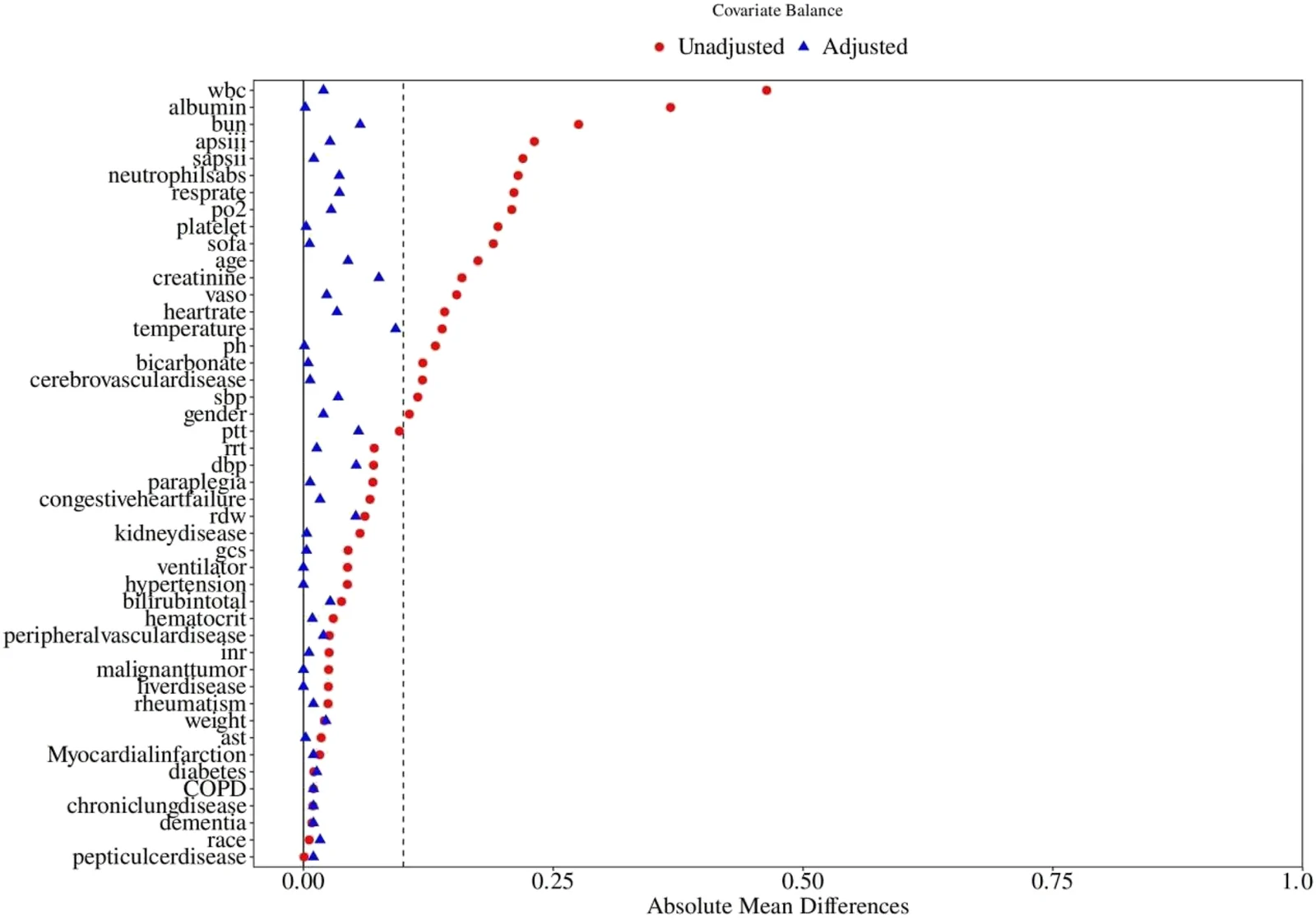
Tendency-based matching of changes in SMD before and after.

### Clinical outcomes

The primary outcome was 28-day all-cause mortality. Secondary outcomes included mortality rates at 60-day, 90-day, 180-day, 1-year, in-ICU, and in-hospital mortality.

### Propensity score matching

Acknowledging the retrospective nature of this study’s design, which inherently poses risks of selection bias and the introduction of confounders, a propensity score matching (PSM) strategy was employed as a corrective measure. Variable selection was based on established clinical relevance and prior sepsis prognostic literature, rather than post-hoc statistical significance. Because many relevant studies have also pointed out the significance of choosing these variables. This approach entailed the formulation of a logistic regression model for the generation of propensity scores, subsequently applied to pair patients in a 1:1 ratio, with a caliper width of 0.05 to ensure precise matching. The effectiveness of PSM was assessed using the standardized mean difference (SMD), with SMD values <0.1 indicating no significant differences. Figure 3 shows the changes in SMD of each indicator before and after PSM. Supplementary Image 3 also illustrates the changes in probability density before and after PSM. C-reactive protein and lymphocyte counts were excluded from the propensity score model because they directly constitute the CLR exposure. Variables with pronounced baseline imbalance (SMD>0.1) before matching – including demographics, vital signs, key laboratory markers, and comorbidities – were closely monitored. Covariate balance after matching was assessed using standardized mean differences, with SMD<0.1 considered adequate.

### Statistical analysis

Continuous variables were presented as median with interquartile range (IQR),and their differences were analyzed using the t-test or Mann-Whitney U-test. Categorical variables were reported as counts with proportions and compared using the Chi-square test or Fisher’s exact test. To determine the optimal cutoff value for the CLR in relation to 28-day mortality, we employed X-tile software (Version 3.6.1, Yale University School of Medicine) that is often used to calculate the optimal cut-off values in survival analysis and continuous data of medical or epidemiological research [13]. By using a complete queue, the optimal cutoff value of 228.5 was determined to maximize the statistical power. To assess internal validity, a training-validation split (50/50) was performed; the training-derived cutoff (228.28) was highly concordant with the full-cohort cutoff (228.5) and demonstrated significant prognostic discrimination in the validation set (log-rank p=0.0002),confirming the robustness of this threshold. For a detailed view, please refer to Supplementary Images 4 and Figure 4. Consequently, CLR was dichotomized into two groups using the pre-determined optimal cutoff value. Graphically, we employed Kaplan–Meier (K–M) curves to visualize the stability of hazard ratios (HRs) in survival analysis. The time-to-event variable in our analysis was the time from ICU admission until the occurrence of the death at endpoint time of interest. The independent association between the CLR and 28-day mortality in septic patients were evaluated using Cox proportional hazard models. The results were reported as HR with 95 % confidence intervals (CI). Three models were constructed: Model 1: Crude. Model 2: Adjust: Gender, RACE, Age, Weight, Heart rate, SBP, DBP, MBP, RR, Temperature, Spo2. Model 3: further adjusted for WBC, Platelet, Neutrophilsabs, Hematocrit, RDW, Bilirubintotal, Creatinine, Bun, Albumin, AST, Bicarbonate, PO2, PH, PTT, INR, APSIII, SAPSII, SOFA score, GCS, Myocardial infarction, congestive heart failure, peripheral vascular disease, cerebrovascular disease, dementia, chronic lung disease, rheumatism, peptic ulcer disease, liver disease, diabetes, paraplegia, kidney disease, malignant tumor, hypertension, COPD. And the corresponding E-valve values were measured as well. We conducted subgroup analyses to investigate the impact of CLR on mortality within different subgroups. Stratification was carried out using a Cox regression model based on age (<65 and ≥65 years), gender, race, comorbidities, treatment.

**Figure 4:**
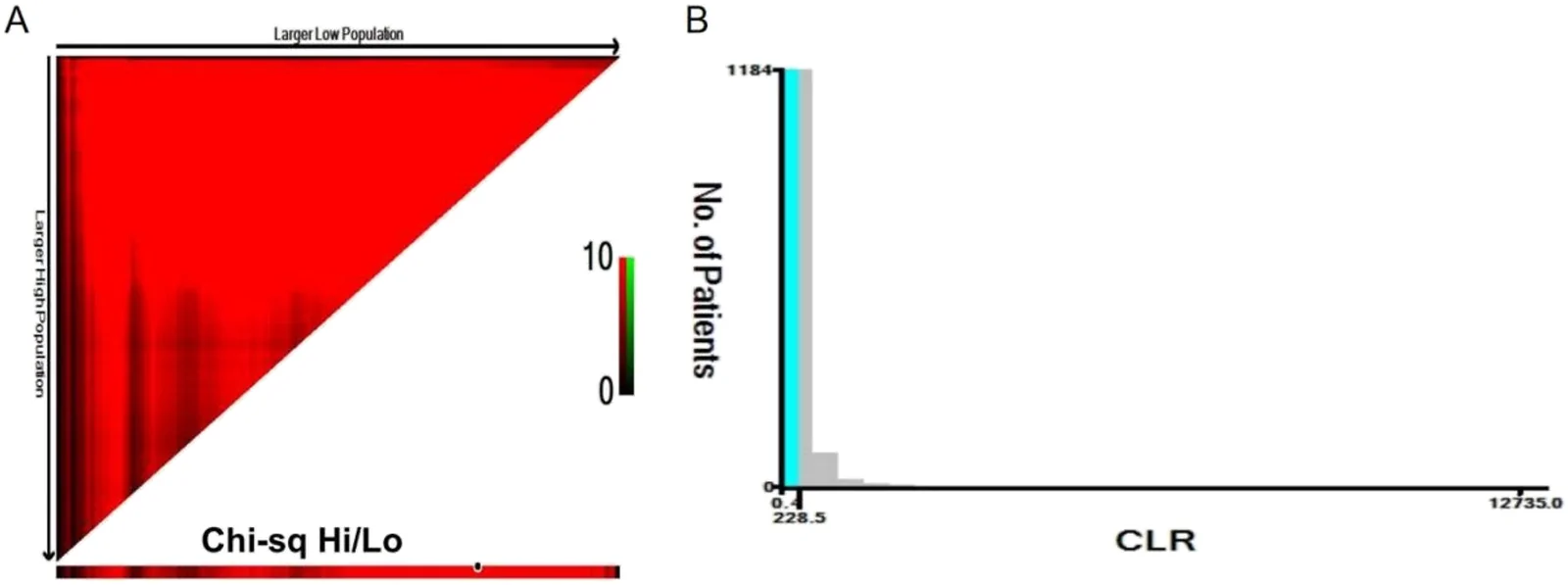
The optimal cutoff value. (A) Histograms. (B) X-tile plots.

To explore potential non-linear relationships, the analysis incorporated restricted cubic splines (RCSs) to achieve more flexible curve fitting. This method aimed to identify any threshold effects and the exact point of inflection for CLR’s influence on mortality in sepsis patients. We select nodes based on the BIC compute and confirmed by initial model diagnostics in our data set. To evaluate the prognostic value of the CLR we performed ROC curve analysis to calculate the area under the curve (AUC) values and compared them using the DeLong-test. We also assessed the incremental predictive value of the CLR by combining it with severity of illness scores, including SAPSII, APSIII, SOFA Score, and GCS. All statistical analyses were two-sided, and p-values less than 0.05 were deemed statistically significant. The software used for analyses included R statistical software (R version 4.2.2, R Foundation for Statistical Computing), SPSS Statistics 26 (IBM, Chicago, IL, USA), and GraphPad Prism 8 (GraphPad Software, San Diego, CA, USA).

## Results

### Baseline characteristics of subjects

Out of the 1,321 patients who met the sepsis-3 criteria and other inclusion criteria for this study. Patients were divided into two groups based on the CLR optimal cutoff value determined by X-tile software, resulting in a low CLR group (<228.5) and a high CLR group (≥228.5), as shown in Figure 4. Before propensity score matching, patients with higher CLR were found to have a higher age and as well as respiratory rate of Ventilator, RRT, Vasoactive drug, and elevated levels of bilirubin total, creatinine, bun, AST, PTT, APSIII, SAPSII, SOFA score, GCS, as shown in Table 1. After the PSM adjustment, it can be observed that the SMD of the dependent variable is all less than 0.1. This indicates that the confounding has been adjusted to a certain extent. For details, please refer to Supplementary Table 2.

**Table 1:** Baseline demographic and clinical characteristics of low-score and high-score groups in the pre-matched cohorts.

Patient Characteristic	Before PSM				
	Total (n=1,321)	Low score (n<228.5) n=988	High score (n≥228.5) n=333	p-Value	SMD
Gender [male, n (%)]	768 (58.14)	548 (55.47)	220 (66.07)	**<0.001**	0.224
Age, years	65.19 (53.71, 74.09)	64.39 (52.20, 73.93)	66.53 (56.90, 74.71)	**0.018**	0.175
Race [white, n (%)]	688 (52.08)	516 (52.23)	172 (51.65)	0.856	−0.012
Weight, kg	82.70 (68.45, 100.00)	82.00 (67.84, 100.70)	84.00 (70.60, 98.00)	0.968	0.021
**Vital signs**					
Heart rate, bpm	91.00 (78.00, 108.00)	90.50 (78.00, 107.00)	93.00 (80.00, 110.00)	0.05	0.141
SBP, mmHg	122.00 (107.00, 140.00)	122.00 (107.00, 141.00)	120.00 (104.00, 137.00)	0.153	−0.114
DBP, mmHg	69.00 (59.00, 81.00)	70.00 (60.00, 82.00)	68.00 (58.00, 80.00)	0.092	−0.07
Respiratory rate, /min	21.00 (17.00, 26.00)	21.00 (17.00, 25.00)	22.00 (18.00, 28.00)	**<0.001**	0.211
Temperature, °C	36.89 (36.56, 37.33)	36.89 (36.56, 37.28)	36.94 (36.61, 37.44)	0.085	0.139
**Laboratory tests**					
WBC (×10^9^)	11.20 (7.80, 16.80)	11.60 (8.30, 17.60)	10.50 (6.60, 15.10)	**<0.001**	−0.464
Platelet (×10^9^)	204.00 (138.00, 281.00)	209.00 (145.00, 286.25)	179.00 (113.00, 261.00)	**<0.001**	−0.195
Neutrophilsabs (×10^9^)	9.56 (6.16, 14.37)	9.71 (6.39, 14.62)	9.11 (5.66, 12.97)	**0.014**	−0.215
Hematocrit, %	33.40 (28.00, 37.80)	33.30 (27.90, 38.00)	33.40 (28.60, 37.40)	0.74	0.03
RDW, %	14.60 (13.40, 16.40)	14.60 (13.40, 16.40)	14.80 (13.70, 16.40)	0.058	0.062
Bilirubintotal, mg/dL	0.60 (0.40, 1.00)	0.60 (0.40, 1.00)	0.60 (0.40, 1.30)	**0.018**	−0.038
Creatinine, mg/dL	1.10 (0.80, 1.90)	1.10 (0.80, 1.80)	1.30 (0.90, 2.20)	**<0.001**	0.159
BUN, mg/dL	24.00 (14.00, 42.00)	22.00 (13.75, 39.25)	29.00 (18.00, 50.00)	**<0.001**	0.275
ALB, g/dL	2.90 (2.50, 3.30)	2.90 (2.50, 3.40)	2.70 (2.40, 3.20)	**<0.001**	−0.367
AST, IU/L	42.00 (25.00, 80.00)	39.00 (24.00, 80.00)	48.00 (30.00, 82.00)	**0.003**	0.018
Bicarbonate, mmol/L	22.00 (19.00, 25.00)	22.00 (20.00, 25.00)	22.00 (19.00, 25.00)	0.05	−0.119
PO2, mmhg	77.00 (49.00, 130.00)	81.00 (49.00, 133.00)	70.00 (46.00, 116.00)	**0.019**	−0.208
PH	7.37 (7.30, 7.43)	7.38 (7.30, 7.43)	7.36 (7.29, 7.42)	**0.029**	−0.132
PTT, s	31.20 (27.40, 38.30)	30.95 (27.20, 37.40)	32.30 (27.80, 40.20)	**0.01**	0.096
INR	1.30 (1.10, 1.60)	1.30 (1.10, 1.50)	1.30 (1.20, 1.60)	0.078	0.026
**Severity of illness scores**					
APSIII	52.00 (39.00, 67.00)	51.00 (38.00, 66.00)	56.00 (42.00, 70.00)	**<0.001**	0.231
SAPSII	39.00 (31.00, 49.00)	38.00 (30.00, 48.00)	41.00 (33.00, 53.00)	**<0.001**	0.22
SOFA score	1.00 (0.00, 3.00)	1.00 (0.00, 3.00)	1.00 (0.00, 4.00)	**0.012**	0.19
GCS	15.00 (15.00, 15.00)	15.00 (14.00, 15.00)	15.00 (15.00, 15.00)	**0.036**	0.045
**Therapy, n (%)**					
Ventilator	1,198 (90.69)	885 (89.57)	313 (93.99)	**0.016**	0.186
RRT	287 (21.73)	197 (19.94)	90 (27.03)	**0.007**	0.16
Vasoactive drug	626 (47.39)	430 (43.52)	196 (58.86)	**<0.001**	0.312
**Comorbidity diseases, n (%)**					
Myocardial infarction	218 (16.5)	159 (16.09)	59 (17.72)	0.49	0.043
Congestive heart failure	438 (33.16)	311 (31.48)	127 (38.14)	**0.026**	0.137
Peripheral vascular disease	149 (11.28)	105 (10.63)	44 (13.21)	0.197	0.076
Cerebrovascular disease	320 (24.22)	269 (27.23)	51 (15.32)	**<0.001**	−0.331
Dementia	63 (4.77)	45 (4.55)	18 (5.41)	0.529	0.038
Chronic lung disease	299 (22.63)	226 (22.87)	73 (21.92)	0.719	−0.023
Rheumatism	59 (4.47)	38 (3.85)	21 (6.31)	0.06	0.101
Peptic ulcer disease	39 (2.95)	29 (2.94)	10 (3.00)	0.95	0.004
Liver disease	223 (16.88)	173 (17.51)	50 (15.02)	0.293	−0.07
Diabetes	430 (32.55)	319 (32.29)	111 (33.33)	0.725	0.022
Paraplegia	148 (11.2)	128 (12.96)	20 (6.01)	**<0.001**	−0.292
Kidney disease	309 (23.39)	217 (21.96)	92 (27.63)	**0.035**	0.127
Malignant tumor	98 (7.42)	67 (6.78)	31 (9.31)	0.128	0.087
Hypertension	468 (35.43)	361 (36.54)	107 (32.13)	0.146	−0.094
COPD	121 (9.16)	93 (9.41)	28 (8.41)	0.583	−0.036

CLR, C-reactive protein to lymphocyte ratio; SBP, Systolic Blood Pressure; DBP, Diastolic Blood Pressure; RR, respiratory rate; WBC, white blood cell; RDW, Red cell volume distribution width; BUN, Blood urea nitrogen; ALB, Albumin; AST, Aspartate Aminotransferase; PO2, Partial pressure of oxygen; PH, Potential of hydrogen; PTT, Partial thromboplastin time; INR, International normalized ratio; GCS, Glasgow coma score; SOFA score, Sequential organ failure assessment score; SAPS-II, Simplified acute physiology score SAPS-II; APS-III, Acute physiology score-III; RRT, Renal replacement treatment; COPD, Chronic obstructive pulmonary disease. Bold values represent all cases where p<0.05.

### The prognosis of sepsis patients in the low-score group and the high-score group

Further description of the results:, before conducting the propensity matching, patients with CLR≥228.5 had a higher risk of various adverse outcomes compared to those in the CLR<228.5 group. They experienced a higher rates of, 28-day mortality (34.38 vs. 21.05 %, p<0.001), 60-day mortality (41.14 vs. 26.82 %, p<0.001), 90-day mortality (43.84 vs. 30.06 %, p<0.001),ICU mortality (29.73 vs. 15.99 %, p<0.001) and in-hospital mortality (36.04 vs. 20.34 %, p<0.001). After performing the PSM matching, the mortality rate of the high-score group was still higher than that of the low-score group. This was specifically manifested in 28-day mortality (33.33 vs. 21.33 %, p<0.001), 60-day mortality (39.33 vs. 28.67 %, p=0.006), 90-day mortality (42.33 vs. 32.33 %, p=0.011), ICU mortality (28.67 vs. 20.33 %, p=0.018), and in-hospital mortality (35 vs. 22.67 %, p<0.001), as shown in Table 2. Notably, while CLR initially appeared correlated with 180-day and 1-year mortality, these associations did not reach statistical significance after PSM (p>0.05).

**Table 2:** Secondary outcomes of septic patients in the low score group and high score group.

Outcomes	Matching	Total	Low-score (<228.5)	High-score (≥228.5)	p-Value
28 day mortality	Before PSM, n (%)	324 (24.53)	208 (21.05)	116 (34.83)	**<0.001**
	After PSM, n (%)	164 (27.33)	64 (21.33)	100 (33.33)	**<0.001**
60 day mortality	Before PSM, n (%)	402 (30.43)	265 (26.82)	137 (41.14)	**<0.001**
	After PSM, n (%)	204 (34.00)	86 (28.67)	118 (39.33)	**0.006**
90 day mortality	Before PSM, n (%)	443 (33.54)	297 (30.06)	146 (43.84)	**<0.001**
	After PSM, n (%)	224 (37.33)	97 (32.33)	127 (42.33)	**0.011**
180 day mortality	Before PSM, n (%)	487 (36.87)	336 (34.01)	151 (45.35)	**<0.001**
	After PSM, n (%)	239 (39.83)	109 (36.33)	130 (43.33)	0.08
1 year mortality	Before PSM, n (%)	545 (41.26)	379 (38.36)	166 (49.85)	**<0.001**
	After PSM, n (%)	268 (44.67)	123 (41.00)	145 (48.33)	0.071
ICU mortality	Before PSM, n (%)	257 (19.45)	158 (15.99)	99 (29.73)	**<0.001**
	After PSM, n (%)	147 (24.50)	61 (20.33)	86 (28.67)	**0.018**
Hospital mortality	Before PSM, n (%)	321 (24.30)	201 (20.34)	120 (36.04)	**<0.001**
	After PSM, n (%)	173 (28.83)	68 (22.67)	105 (35.00)	**<0.001**

Bold values represent all cases where p<0.05.

The Kaplan–Meier survival curve also indicates that before and after PSM, the 28-day mortality rate in the Low-score group was lower (log-rank test: p<0.001; Figure 5). Results from the 60 day and 90 day and in-hospital survival and in-ICU curves were consistent with those from the 28 day results (detailed results are available in Supplementary Images 5, 6, 7 and 8).

**Figure 5:**
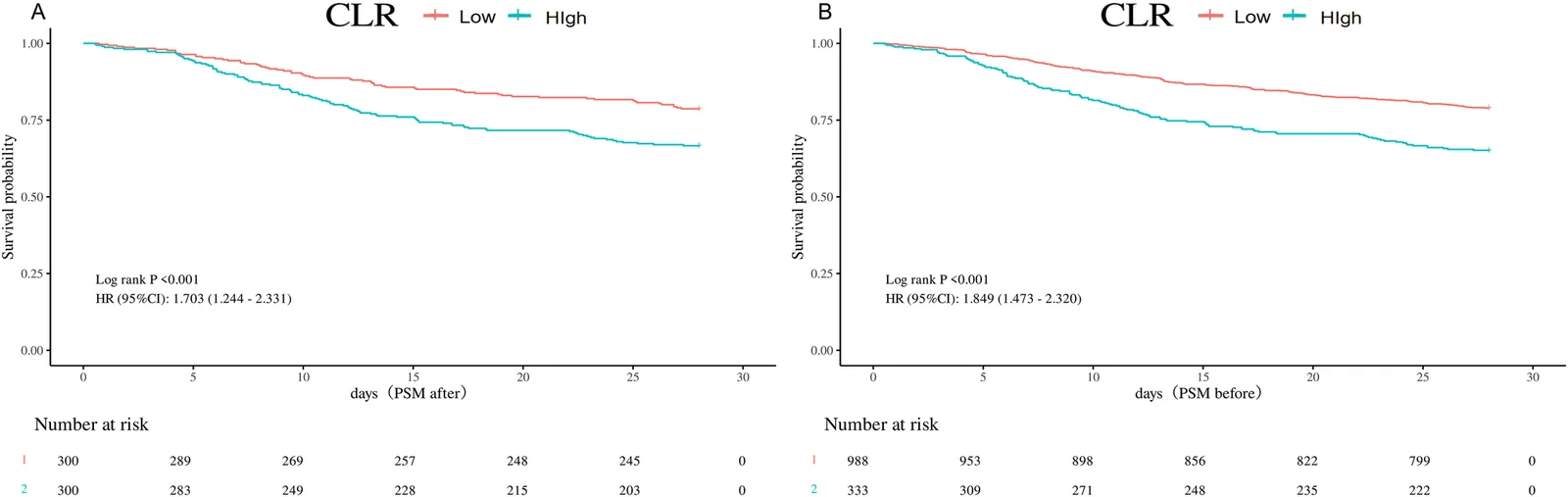
Kaplan–Meier survival curves for 28-day mortality in sepsis patients from the low group and the high group. (A) Before PSM. (B) After PSM.

These results collectively indicate that patients with sepsis with a CLR score lower than 228.5 have a higher survival rate, suggesting that this score could be a potential indicator for prognostic risk stratification. The clinical application value of this score awaits further verification with a larger sample in the future.

### Cox regression analyses evaluating the relationship between CLR and prognosis in sepsis patients before PSM

To evaluate the potential relationship between CLR and mortality outcomes in patients with sepsis, both univariate and multivariate Cox proportional hazard models were executed with CLR dichotomized at the threshold of 228.5. In the initial crude model (Model 1), a heightened CLR (≥228.5) was demonstrably linked with an escalated risk of 28-day mortality (HR=1.85, 95 % CI: 1.47–2.32, p<0.001). This association remained statistically significant in Model 2 after adjusting for demographic variables and vital signs (HR=1.69, 95 % CI: 1.34–2.13, p<0.001). Even in the fully adjusted model (Model 3), which accounted for laboratory parameters, comorbidities, and established severity scores – including SOFA, SAPS II, APS III, and GCS – the high CLR group persisted in showing an independent and elevated risk for mortality (HR=1.55, 95 % CI: 1.22–1.99, p<0.001). This confirms that an elevated CLR is independently associated with a poor short-term prognosis. It can be clearly seen in Table 3.

**Table 3:** Cox proportional hazard models for 28 day mortality.

Variables	Model1		Model2		Model3		E-valve E-valve (95 % CI)	
	HR (95 % CI)	p-Value	HR (95 % CI)	p-Value	HR (95 % CI)	p-Value		
**CLR dichotomous**								
Low score (<228.5)	Reference		Reference		Reference			
High score (≥228.5)	1.85 (1.47–2.32)	**<0.001**	1.69 (1.34–2.13)	**<0.001**	1.55 (1.22–1.99)	**<0.001**	2.47	(1.74–3.39)

HR, hazard ratio; CI, confidence interval. Model 1: Crude. Model 2: adjust: gender, race, age, weight, heartrate, SBP, DBP, respiratory rate, temperature. Model 3: Adjust: Model 2+WBC, Platelet, Neutrophilsabs, Hematocrit, RDW, Bilirubintotal, Creatinine, Bun, ALB, AST, Bicarbonate, PO2, PH, PTT, INR, APSIII, SAPSII, SOFA score, GCS, Myocardial infarction, congestive heart failure, peripheral vascular disease, cerebrovascular disease, dementia, chronic lung disease, rheumatism, peptic ulcer disease, liver disease, diabetes, paraplegia, kidney disease, malignant tumor, hypertension, COPD. CLR, C-reactive protein to lymphocyte ratio; SBP, Systolic Blood Pressure; DBP, Diastolic Blood Pressure; RR, respiratory rate; WBC, white blood cell; RDW, Red cell volume distribution width; BUN, Blood urea nitrogen; ALB, Albumin; AST, Aspartate Aminotransferase; PO2, Partial pressure of oxygen; PH, Potential of hydrogen; PTT, Partial thromboplastin time; INR, International normalized ratio; GCS, Glasgow coma score; SOFA score, Sequential organ failure assessment score; SAPS-II, Simplified acute physiology score SAPS-II; APS-III, Acute physiology score-III; COPD, Chronic obstructive pulmonary disease. Bold values represent all cases where p<0.05.

A sensitivity analysis was further conducted to assess the stability of these findings and to address the potential confounding or mediating effects of clinical interventions on mortality. Specifically, Model 4 incorporated major therapeutic measures – including mechanical ventilation, renal replacement therapy (RRT), and the administration of vasopressors – into the variables already adjusted in Model 3. For a detailed view, please refer to Supplementary Table 3. Despite the addition of these factors, the 28-day mortality risk in the high CLR group remained stable (HR=1.51, 95 % CI: 1.18–1.93, p<0.001), reinforcing the conclusion that the prognostic value of CLR is independent of key supportive treatments. Furthermore, to evaluate the potential impact of unmeasured confounding, we calculated the E-value. For 28-day mortality, the E-value was 2.47 (95 % CI: 1.74–3.39), indicating that an unmeasured confounder would require a substantial effect size to negate the observed association between CLR and death, thereby confirming the robustness of the results.

### Subgroup analysis for the CLR on clinical outcomes in patients with sepsis

Figure 6 presents the forest plot of subgroup analyses examining the association between the CLR and 28-day mortality. Overall, a high CLR was significantly associated with increased 28-day mortality (HR 1.70, 95 % CI: 1.24–2.33, p<0.001). This association remained consistent across most subgroups, including gender, race, and comorbidities such as congestive heart failure, chronic lung disease, and diabetes. Notably, the association was stronger in patients aged ≥65 years (HR 2.19, 95 % CI: 1.49–3.22, p<0.001), those not on ventilators (HR 5.21, 95 % CI: 1.12–24.17, p=0.035), and those with diabetes (HR 2.25, 95 % CI: 1.50–3.39, p<0.001). Additionally, significant interactions were observed for age (p for interaction=0.045), diabetes (p for interaction=0.025), and paraplegia (p for interaction=0.034), suggesting that the prognostic value of CLR may vary across these specific subgroups. Furthermore, while a particularly strong association was observed between CLR and mortality in the non-mechanically ventilated subgroup, this finding should be interpreted with caution. The total number of patients in the non-mechanical ventilation group was approximately 40, which tended to lead to unstable results. More multi-center studies will still be needed in the future to verify this judgment.

**Figure 6:**
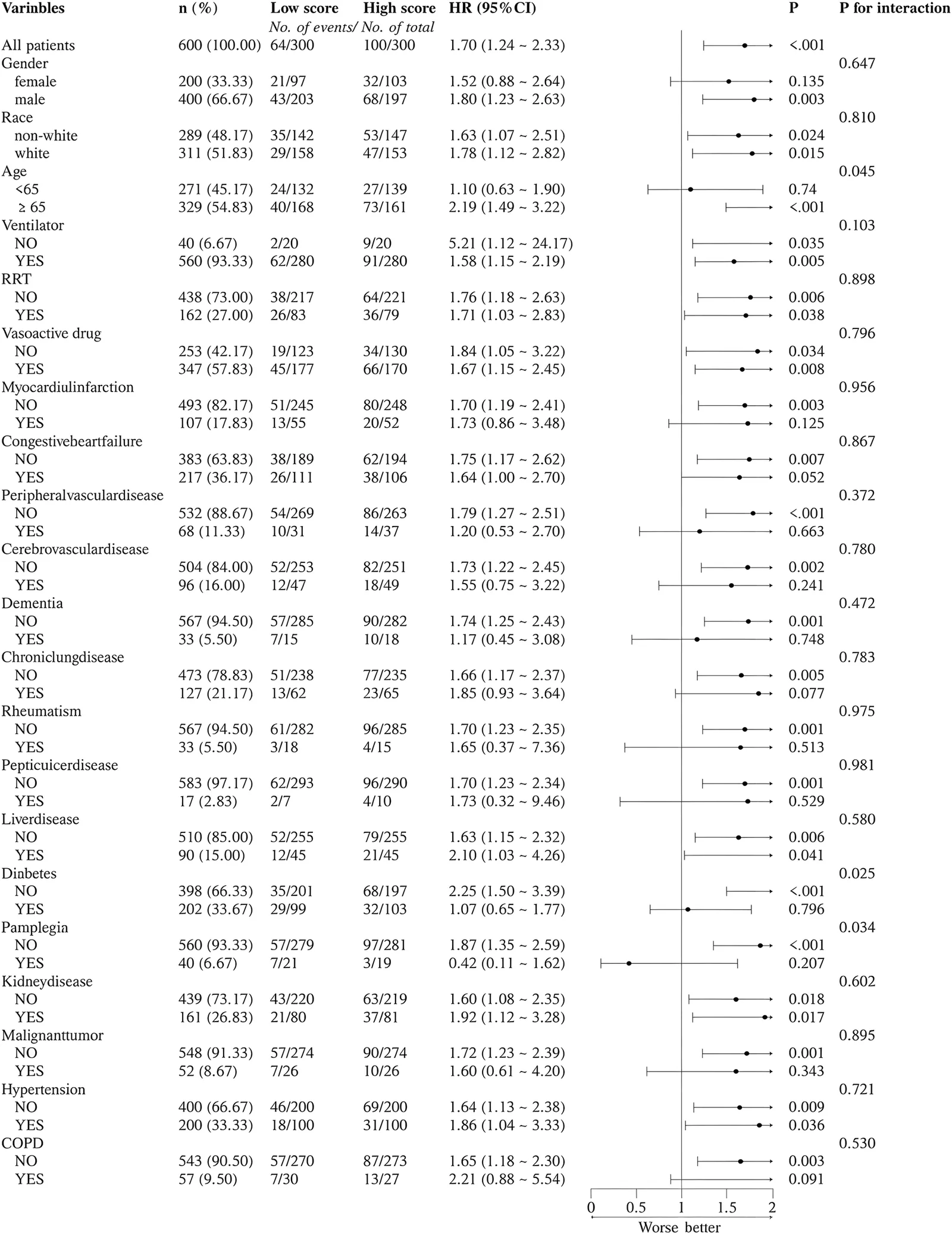
Subgroup analysis of the relationship between CLR and 28-day mortality, as visualized by a forest plot.

### Nonlinear relationship of CLR and all cause mortality of sepsis patient

Figure 7 uses a constrained cubic spline model to illustrate the relationship between CLR and 28-day mortality in patients with sepsis. The covariates included are the same as those used in the PSM, such as basic vital signs, laboratory tests, and comorbidities. The analysis revealed a significant nonlinear association (p for overall=0.001, p for nonlinear=0.004), with a clear inflection point identified at a CLR value of 228.5. Below this threshold, mortality risk increased gradually with rising CLR; however, beyond 228.5, the risk escalated more sharply, indicating a critical threshold effect. These findings suggest that CLR levels above 228.5 are associated with a substantially higher risk of 28-day mortality, highlighting its potential as a prognostic biomarker in sepsis management.

**Figure 7:**
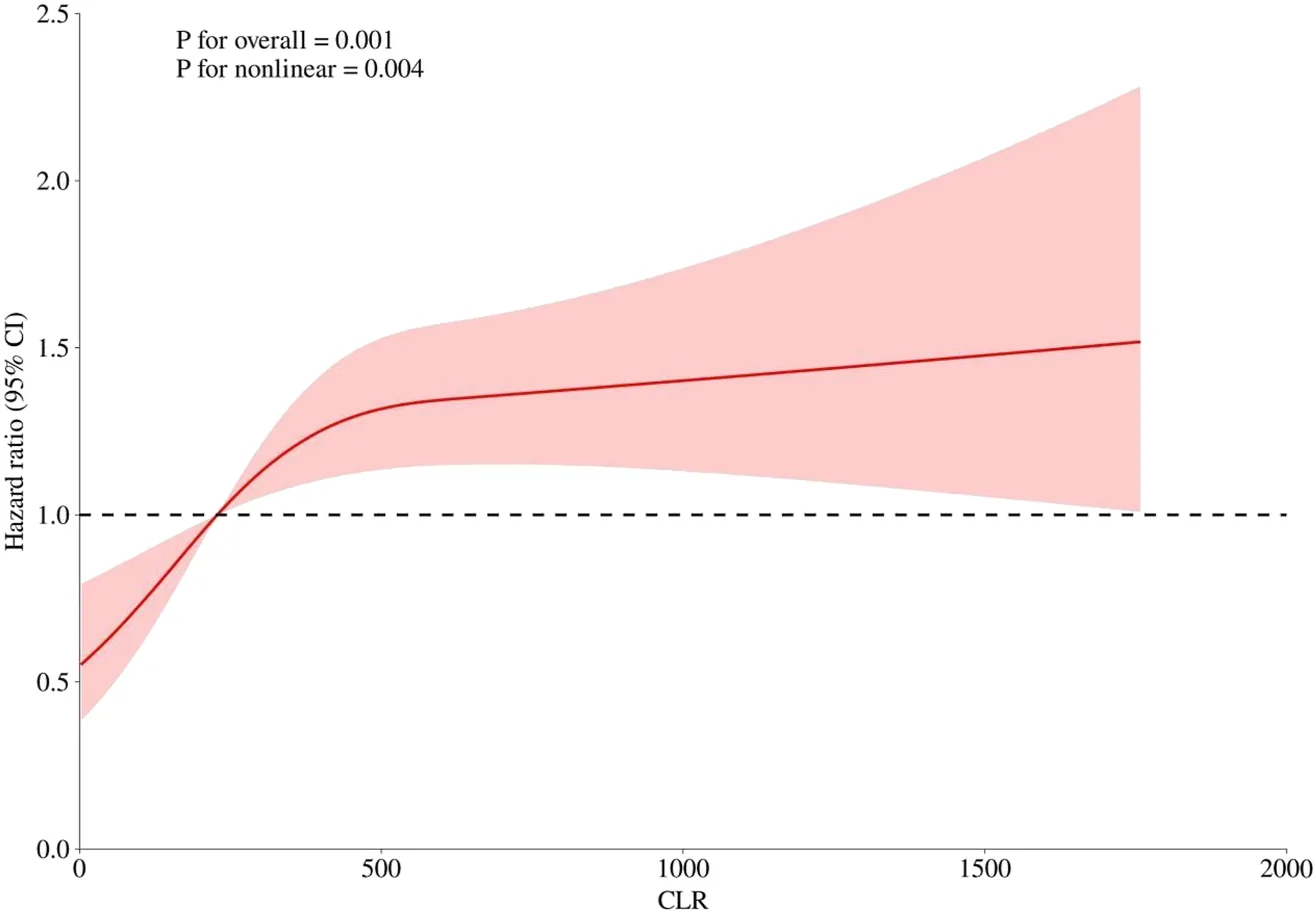
The restricted cubic spline analysis of the CLR and 28-day mortality in patients with sepsis.

### Prognostic value of CLR and severity of illness scores

Table 4 presents the predictive performance of CLR alone and in combination with established severity scores for 28-day mortality. The AUC for CLR alone was 0.58. When combined with SOFA, APS III, SAPS II, and GCS scores, the AUCs improved slightly to 0.59, 0.67, 0.68, and 0.59, respectively. Notably, the addition of CLR significantly enhanced the predictive accuracy of each model (all p<0.05), indicating that CLR provides incremental prognostic value beyond traditional scoring systems.

**Table 4:** The area under the curve (AUC) for predicting 28-day mortality by CLR and CLR combined with other models.

Variables	AUC 95 % CI	p-Value
CLR	0.58 (0.55–0.62)	
SOFA score	0.55 (0.51–0.59)	
SOFA score + CLR	0.59 (0.55–0.62)	<0.001^a^
APSIII	0.66 (0.63–0.69)	
APSIII + CLR	0.67 (0.63–0.70)	0.024^b^
SAPSII	0.67 (0.64–0.70)	
SAPSII + CLR	0.68 (0.65–0.71)	<0.001^c^
GCS	0.53 (0.50–0.56)	
GCS + CLR	0.59 (0.56–0.63)	<0.001^d^

^a^Represent the statistical significance of the SOFA score + CLR in AUC and the SOFA score. ^b^Represent the statistical significance of the APSIII + CLR in AUC and the APSIII. ^c^Represent the statistical significance of the SAPSII + CLR in AUC and the SAPSII. ^d^Represent the statistical significance of GCS + CLR in AUC and GCS. CLR, C-reactive protein to lymphocyte ratio; GCS, Glasgow coma score; SOFA score, Sequential organ failure assessment score; SAPS-II, Simplified acute physiology score SAPS-II; APS-III, Acute physiology score-III.

## Discussion

By retrospective analysis of 1,321 patients with sepsis in the MIMIC-IV database, this study revealed for the first time the non-linear relationship between the C-reactive protein-to-lymphocyte ratio (CLR) and the prognosis of sepsis. We found that patients with CLR≥228.5 had significantly increased mortality across 28-day, 60-day, 90-day, ICU, and hospital stay periods. Notably, while CLR initially appeared correlated with 180-day and 1-year mortality, these associations did not reach statistical significance after propensity score matching (PSM) (p>0.05). This divergence indicates that CLR serves as a more specific and potent marker for short-term risk stratification during the acute phase of sepsis, rather than a long-term prognostic indicator.

Previous studies have confirmed the correlation between various inflammatory biomarkers – including CRP, procalcitonin (PCT), and complex indices like the neutrophil-to-lymphocyte ratio (NLR), platelet-to-lymphocyte ratio (PLR), and systemic immune-inflammation index (SII) – and sepsis outcomes [14], [15], [16], [17]. In our study, CLR exhibited a substantial independent risk association for 28-day mortality with a hazard ratio (HR) of 1.51, a magnitude that is highly competitive with reported effect sizes for NLR (often cited with ORs around 1.28 in similar cohorts) [18]. While CRP reflects the magnitude of the systemic inflammatory response and serves as a reliable indicator of initial severity, the lymphocyte count reflects immune dysfunction and exhaustion [19], 20]. By integrating these two parameters, CLR effectively captures the “inflammation-immunosuppression” balance, providing a critical real-time snapshot of the patient’s status that appears more robust than single-parameter indicators.

The prognostic value of CLR is not limited to sepsis; it demonstrates significant “horizontal” utility across various inflammatory conditions. Extensive research has shown that elevated CLR is an independent risk factor for poor outcomes in multiple diseases, including certain cancers and systemic inflammatory disorders such as COVID-19 [21], [22], [23], [24]. Similarly, CLR has been validated as a powerful predictor of adverse outcomes in acute pancreatitis and myocardial infarction [25], 26]. In the context of sepsis, we observed a distinct threshold effect at 228.5 through restricted cubic spline (RCS) analysis, above which the mortality risk increased sharply. This non-linear characterization allows for more precise risk stratification compared to linear scoring. Although the standalone AUC of CLR was modest (0.58), its integration with established severity scores effectively enhanced their predictive performance. As an auxiliary tool alongside standard diagnostic procedures like blood gas analysis, CLR facilitates the early identification of high-risk patients, enabling timely interventions that are essential for improving patient outcomes.

In our subgroup analyses, significant interactions were observed between CLR and clinical variables such as age and diabetes. The enhanced prognostic value in elderly patients may be attributed to “inflammaging” – baseline immune dysfunction and chronic inflammatory status [27]. Similarly, in the diabetes subgroup, hyperglycemia may exacerbate the threshold effect by activating the Mitochondrial apoptosis and intensifying lymphocyte apoptosis [28]. Furthermore, while a particularly strong association was observed between CLR and mortality in the non-mechanically ventilated subgroup, this finding should be interpreted with caution. The potentially limited sample size in this sub-population may lead to statistically unstable estimates, necessitating verification in larger cohorts.

Several limitations must be acknowledged. Although we employed rigorous statistical techniques, including Cox proportional hazards models, multiple imputation, and PSM, residual confounding cannot be entirely eliminated. To further assess the robustness of our findings, E-value analysis was performed, quantifying the strength required for an unmeasured confounder to negate our results. However, certain key variables, such as PCT and IL-6, were partially missing in the database, which may limit the depth of our inflammatory profile analysis. Additionally, CLR is intended as an auxiliary indicator that complements primary assessments like arterial blood gas analysis. Future research should prioritize multicenter cohorts and large-scale prospective studies to validate the cross-population effectiveness of CLR and further explore its prognostic value in specific high-risk subpopulations.

## Conclusions

Our study found that CLR is independently associated with increased short-term mortality in sepsis patients. CLR serves as a readily available prognostic indicator for risk stratification and may facilitate early clinical intervention to improve patient outcomes.

## Supplementary Material

Supplementary Material

Supplementary Material

Supplementary Material

Supplementary Material

Supplementary Material

Supplementary Material

Supplementary Material

Supplementary Material

Supplementary Material

Supplementary Material

Supplementary Material
