# Supplementary material for: The C-reactive protein to lymphocyte ratio and 28-day mortality in critically ill patients with sepsis: a retrospective propensity score–matched cohort study
Source: Open Med (Wars). 2026 Nov 11;21(1):20261548. doi: 10.1515/med-2026-1548 (PMC13749928; doi:10.1515/med-2026-1548)
Supplement: Supplementary file 1 [file j_med-2026-1548_suppl_009.docx]

| Supplementary Table 1: The missing variables are due to a missing value rate greater than 20% or multicollinearity. | |
| --- | --- |
| The data is missing by more than 20% | Height(26.1%),BMI(26.2%),Calcium(30.8%),Chloride(75.3%),Glucose(49%),Sodium(65.7%),Potassium(48.2%),Hemoglobin(72.5%),Ck(37.3%),CKMB(49.3%),fio2(46.1%),pao2fio2ratio(23.8%),Cholesterol(82.2%),Troponin(56.3%),BNP(84.5%) |
| VIF is greater than 5 or the tolerance is less than 0.1 | ALT,MBP,PT,CII,baseexcess |
