# Supplementary material for: The C-reactive protein to lymphocyte ratio and 28-day mortality in critically ill patients with sepsis: a retrospective propensity score–matched cohort study
Source: Open Med (Wars). 2026 Nov 11;21(1):20261548. doi: 10.1515/med-2026-1548 (PMC13749928; doi:10.1515/med-2026-1548)
Supplement: Supplementary file 2 [file j_med-2026-1548_suppl_010.docx]

| Supplementary Table 2 Baseline demographic and clinical characteristics of Low-score and High-score groups in the post-matched cohorts | | | | | |
| --- | --- | --- | --- | --- | --- |
|  |  |  |  |  |  |
| Patient | After PSM | | | | |
| characteristic | Total (n = 600) | Low score(n<228.5)n = 300 | High score (n≥228.5)n = 300 | *P* | SMD |
| Gender[male, n (%)] | 400 (66.67) | 203 (67.67) | 197 (65.67) | 0.603 | -0.042 |
| Age(years) | 66.55 (56.11, 75.44) | 67.72 (56.19, 76.42) | 65.93 (56.01, 73.99) | 0.3 | -0.044 |
| Race[white, n (%)] | 311 (51.83) | 158 (52.67) | 153 (51.00) | 0.683 | -0.033 |
| Weight(kg) | 83.35 (69.92, 99.55) | 82.60 (68.83, 100.73) | 84.50 (70.97, 98.12) | 0.967 | 0.022 |
| Vital signs |  |  |  |  |  |
| Heartrate(bpm) | 93.00 (79.00, 110.25) | 94.00 (79.75, 112.25) | 92.00 (79.00, 109.25) | 0.631 | -0.035 |
| SBP(mmHg) | 119.00 (105.00, 138.00) | 119.00 (106.00, 138.25) | 120.00 (104.00, 137.00) | 0.905 | -0.034 |
| DBP(mmHg) | 69.00 (59.00, 81.00) | 69.00 (59.00, 81.00) | 68.00 (59.00, 80.25) | 0.487 | -0.055 |
| Respiratory rate (/min) | 22.00 (18.00, 26.00) | 22.00 (18.00, 26.00) | 22.00 (18.00, 27.00) | 0.666 | 0.036 |
| Temperature(°C) | 36.89 (36.61, 37.39) | 36.89 (36.56, 37.39) | 36.89 (36.61, 37.40) | 0.521 | 0.097 |
| Laboratory tests |  |  |  |  |  |
| WBC(×10^9^) | 10.60 (7.10, 15.20) | 10.45 (7.50, 14.93) | 10.65 (6.80, 15.50) | 0.867 | -0.02 |
| Platelet(×10^9^) | 189.50 (126.00, 266.00) | 190.50 (133.75, 260.00) | 187.00 (123.00, 268.25) | 0.664 | 0.003 |
| Neutrophilsabs(×10^9^) | 9.21 (5.87, 13.72) | 9.07 (6.00, 13.78) | 9.27 (5.79, 13.36) | 0.804 | -0.036 |
| Hematocrit(%) | 33.70 (28.37, 37.92) | 33.90 (28.25, 38.42) | 33.35 (28.58, 37.42) | 0.626 | -0.009 |
| RDW(%) | 14.60 (13.50, 16.50) | 14.60 (13.40, 16.50) | 14.75 (13.67, 16.40) | 0.249 | 0.052 |
| Bilirubintotal(mg/dL) | 0.60 (0.40, 1.10) | 0.60 (0.40, 1.00) | 0.60 (0.40, 1.20) | 0.261 | 0.026 |
| Creatinine(mg/dL) | 1.20 (0.80, 2.10) | 1.20 (0.80, 2.20) | 1.20 (0.80, 2.00) | 0.677 | 0.073 |
| Bun(mg/dL) | 27.00 (16.00, 46.00) | 25.00 (15.00, 45.00) | 29.00 (17.00, 48.00) | 0.164 | 0.059 |
| Albumin(g/dL) | 2.80 (2.40, 3.20) | 2.80 (2.40, 3.20) | 2.80 (2.40, 3.20) | 0.993 | 0.002 |
| AST(IU/L) | 46.00 (28.00, 88.25) | 44.00 (26.00, 94.25) | 48.00 (29.75, 85.25) | 0.463 | -0.002 |
| Bicarbonate(mmol/L) | 22.00 (19.00, 25.00) | 22.00 (19.00, 25.00) | 22.00 (19.00, 25.00) | 0.699 | 0.005 |
| PO2(mmhg) | 75.00 (48.00, 119.25) | 77.00 (46.00, 118.00) | 72.00 (49.00, 121.25) | 0.974 | -0.028 |
| PH | 7.37 (7.29, 7.42) | 7.37 (7.29, 7.42) | 7.37 (7.29, 7.42) | 0.804 | 0.001 |
| PTT(sec) | 32.05 (28.10, 40.20) | 32.05 (28.48, 40.23) | 32.05 (27.50, 40.05) | 0.341 | -0.055 |
| INR | 1.30 (1.20, 1.60) | 1.30 (1.20, 1.60) | 1.30 (1.20, 1.60) | 0.965 | 0.005 |
| Severity of illness scores |  |  |  |  |  |
| APSIII | 54.00 (41.00, 68.00) | 53.00 (40.75, 69.00) | 55.00 (41.75, 68.00) | 0.637 | 0.027 |
| SAPSII | 41.00 (32.00, 51.00) | 41.00 (32.00, 51.00) | 40.50 (32.00, 52.00) | 0.949 | 0.01 |
| SOFA score | 1.00 (0.00, 4.00) | 1.00 (0.00, 4.00) | 1.00 (0.00, 4.00) | 0.621 | 0.006 |
| GCS | 15.00 (15.00, 15.00) | 15.00 (15.00, 15.00) | 15.00 (15.00, 15.00) | 0.182 | 0.003 |
| Therapy, n(%) |  |  |  |  |  |
| Ventilator | 560 (93.33) | 280 (93.33) | 280 (93.33) | 1 | 0 |
| RRT | 162 (27) | 83 (27.67) | 79 (26.33) | 0.713 | 0.03 |
| Vasoactive drug | 347 (57.83) | 177 (59.00) | 170 (56.67) | 0.563 | 0.047 |
| Comorbidity diseases, n(%) |  |  |  |  |  |
| Myocardialinfarction | 107 (17.83) | 55 (18.33) | 52 (17.33) | 0.749 | -0.026 |
| Congestiveheartfailure | 217 (36.17) | 111 (37.00) | 106 (35.33) | 0.671 | -0.035 |
| Peripheralvasculardisease | 68 (11.33) | 31 (10.33) | 37 (12.33) | 0.44 | 0.061 |
| Cerebrovasculardisease | 96 (16) | 47 (15.67) | 49 (16.33) | 0.824 | 0.018 |
| Dementia | 33 (5.5) | 15 (5.00) | 18 (6.00) | 0.591 | 0.042 |
| Chroniclungdisease | 127 (21.17) | 62 (20.67) | 65 (21.67) | 0.764 | 0.024 |
| Rheumatism | 33 (5.5) | 18 (6.00) | 15 (5.00) | 0.591 | -0.046 |
| Pepticulcerdisease | 17 (2.83) | 7 (2.33) | 10 (3.33) | 0.46 | 0.056 |
| Liverdisease | 90 (15) | 45 (15.00) | 45 (15.00) | 1 | 0 |
| Diabetes | 202 (33.67) | 99 (33.00) | 103 (34.33) | 0.73 | 0.028 |
| Paraplegia | 40 (6.67) | 21 (7.00) | 19 (6.33) | 0.743 | -0.027 |
| Kidneydisease | 161 (26.83) | 80 (26.67) | 81 (27.00) | 0.927 | 0.008 |
| Malignanttumor | 52 (8.67) | 26 (8.67) | 26 (8.67) | 1 | 0 |
| Hypertension | 200 (33.33) | 100 (33.33) | 100 (33.33) | 1 | 0 |
| COPD | 57 (9.5) | 30 (10.00) | 27 (9.00) | 0.676 | -0.035 |
