# Supplementary material for: The C-reactive protein to lymphocyte ratio and 28-day mortality in critically ill patients with sepsis: a retrospective propensity score–matched cohort study
Source: Open Med (Wars). 2026 Nov 11;21(1):20261548. doi: 10.1515/med-2026-1548 (PMC13749928; doi:10.1515/med-2026-1548)
Supplement: Supplementary file 3 [file j_med-2026-1548_suppl_011.docx]

| Supplementary Table 3: Further adjusting the variables of the treatment plan based on the model 3 results | | |
| --- | --- | --- |
| Variables | Model4 | |
| CLR dichotomous | | |
|  | HR(95%CI) | P-valve |
| low score(<228.5) | Reference | |
| high score(≥228.5) | 1.51 (1.18 ~ 1.93) | **<.001** |
